# Supplementary figures and images for: Response of oat morphologies, root exudates, and rhizosphere fungal communities to amendments in a saline-alkaline environment
Source: PLoS One. 2020 Dec 3;15(12):e0243301. doi: 10.1371/journal.pone.0243301 (PMC7714365; doi:10.1371/journal.pone.0243301)

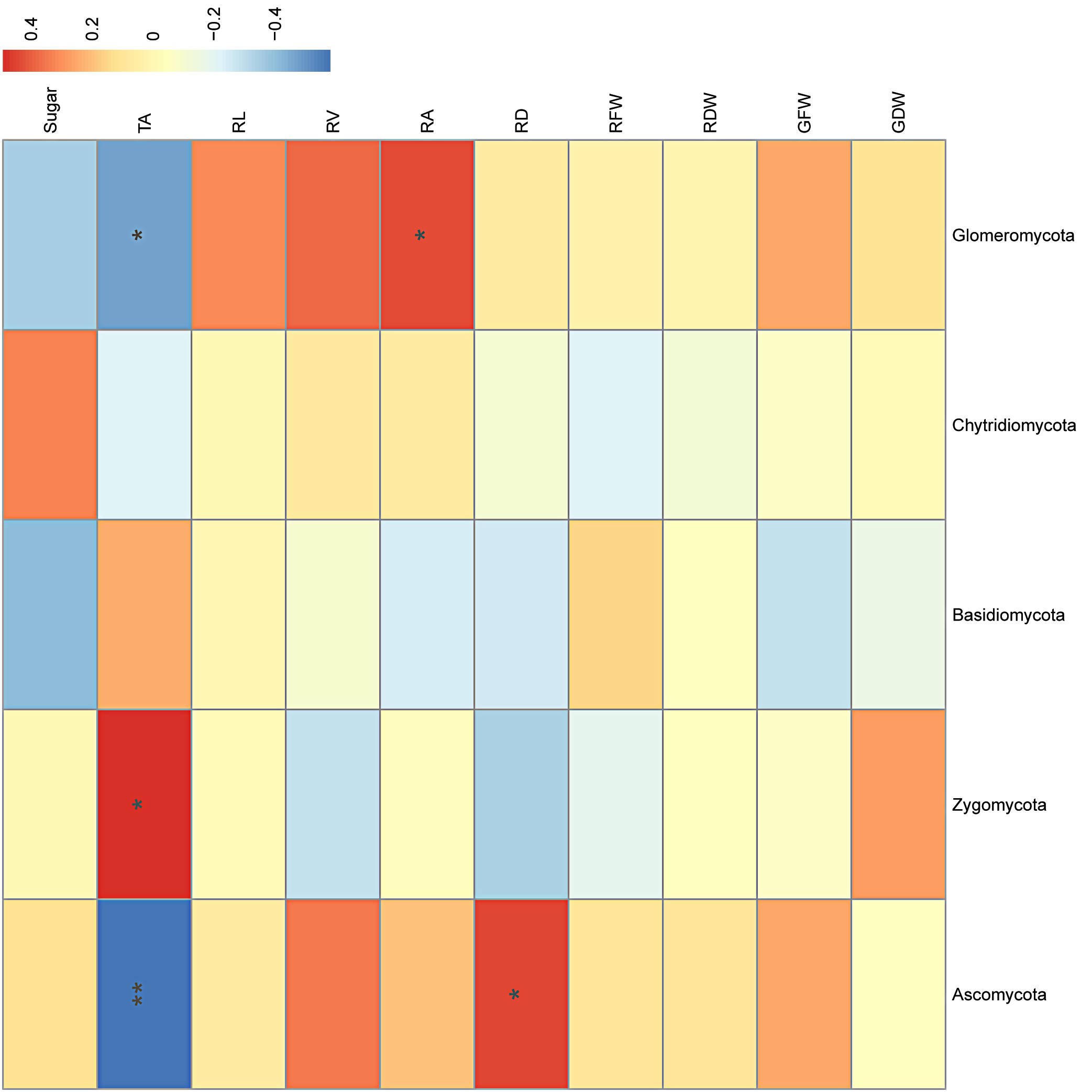

Supplement: S1 Fig — An r < 0 indicates a negative correlation and an r > 0 indicates a positive correlation. The labels *, ** and *** indicate significance levels of p < 0.05, p < 0.01 and p < 0.001, respectively. The following parameters were used: sugar, soluble sugar; TA, total organic acid; RL, root length; RV, root volume; RA, root surface area; RD, root diameter; RFW, root fresh biomass; RDW, root dry biomass; GFW, shoot fresh biomass; and GDW, shoot dry biomass. (TIF) [file pone.0243301.s001.tif]
